# Electrospun Scaffolds Enriched with Nanoparticle-Associated DNA: General Properties, DNA Release and Cell Transfection

**DOI:** 10.3390/polym15153202

**Published:** 2023-07-27

**Authors:** Vera Chernonosova, Marianna Khlebnikova, Victoriya Popova, Ekaterina Starostina, Elena Kiseleva, Boris Chelobanov, Ren Kvon, Elena Dmitrienko, Pavel Laktionov

**Affiliations:** 1 Institute of Chemical Biology and Fundamental Medicine, Siberian Branch, Russian Academy of Sciences, 630090 Novosibirsk, Russia; m.khlebnikova@g.nsu.ru (M.K.); fomenkoniboch@gmail.com (V.P.); boris.p.chelobanov@gmail.com (B.C.); elenad@niboch.nsc.ru (E.D.); 2 State Research Center of Virology and Biotechnology VECTOR, Rospotrebnadzor, 630559 Koltsovo, Russia; starostina_ev@vector.nsc.ru; 3 Institute of Cytology and Genetics, Siberian Branch, Russian Academy of Sciences, 630090 Novosibirsk, Russia; elka@bionet.nsc.ru; 4 Boreskov Institute of Catalysis, Siberian Branch, Russian Academy of Sciences, 630090 Novosibirsk, Russia; kvon@catalysis.ru

**Keywords:** electrospun scaffold, DNA delivery, plasmid DNA, nanoparticle, DNA release, cell transfection, manipulating cellular behaviors

## Abstract

Biomaterial-mediated, spatially localized gene delivery is important for the development of cell-populated scaffolds used in tissue engineering. Cells adhering to or penetrating into such a scaffold are to be transfected with a preloaded gene that induces the production of secreted proteins or cell reprogramming. In the present study, we produced silica nanoparticles-associated pDNA and electrospun scaffolds loaded with such nanoparticles, and studied the release of pDNA from scaffolds and cell-to-scaffold interactions in terms of cell viability and pDNA transfection efficacy. The pDNA-coated nanoparticles were characterized with dynamic light scattering and transmission electron microscopy. Particle sizes ranging from 56 to 78 nm were indicative of their potential for cell transfection. The scaffolds were characterized using scanning electron microscopy, X-ray photoelectron spectroscopy, stress-loading tests and interaction with HEK293T cells. It was found that the properties of materials and the pDNA released vary, depending on the scaffold’s composition. The scaffolds loaded with pDNA-nanoparticles do not have a pronounced cytotoxic effect, and can be recommended for cell transfection. It was found that (pDNA-NPs) + PEI9-loaded scaffold demonstrates good potential for cell transfection. Thus, electrospun scaffolds suitable for the transfection of inhabiting cells are eligible for use in tissue engineering.

## 1. Introduction

Tissue engineering suggests the production of an analog of native tissue using artificial scaffolds that is able to be seeded by cells. Taking into account that natural tissue consists of many cell types located in different tissue compartments depending on tissue topology, the guidance and long-term maintenance of compartment-specific cell phenotypes are needed, and represent a complicated task. This task is at least no less Important than the maintenance of 3D structure and mechanical properties of artificial tissue, which is required for the integration and long-term functioning of tissue-engineered constructs [1,2,3]. It should be mentioned that the delivery of specific molecules in tissue compartments is feasible due to the mode of 3D scaffold production like 3D printing or other additive technologies [3,4,5,6] that enable the introduction of molecules, depending on the 3D scaffold’s location during production. The introduction of special reagents inside scaffolding has obvious advantages over systemic delivery, since this approach could provide local delivery, require much fewer reagents and escape side effects due to their extra low concentrations required in systemic circulation.

Artificial construct/scaffolds may be seeded by cells in vitro in bioreactors; such a mode of cell seeding does not automatically solve the problem of definite cell type location in the construct [7,8]. Being free or seeded by cells, after implantation, scaffolds contact surrounding cells and may be seeded by these cells (replace already adhered cells as well); thus, guidance in terms of their phenotype is also required.

It should be mentioned that implanted scaffolds, in any way, must inhibit inflammation induced through surgery as well as foreign body reactions, and must initiate colonization of the scaffold with cells of the required phenotype [9,10]. To inhibit inflammation, small-molecule drugs could be used, along with anti-inflammatory proteins like IL4 or IL10 [9,11]. In this respect, one must note that the acute phase of inflammation is short, and the delivery of such molecules may be realized by published methods [10,12]. In contrast, support of long-term protein secretions or cell reprogramming takes time, and must be controlled.

In this respect, the long-term delivery of protein growth factors is limited due to denaturation related to storage and bioactivity loss [13,14]. The use of mRNAs or small RNAs for cell type guidance is also complicated by their stability and/or multi-target miRNA nature [15].

In contrast to the aforementioned, DNA vectors encoding desired molecules (transcription factors, interleukins, cell migration activators, etc.) are stable molecules that could be deposited and released for a long time [15,16]. Actually, the delivery of coding DNA into cells allows for both direct influence on the cell phenotype [17,18] and induction of the expression of growth factors, cytokines and chemo attractants, etc. [19,20,21].

Returning to the production of the scaffolds, we cannot forget that electrospinning (ES) and the scaffolds produced by this method could be used for soft and hard tissue engineering [22,23]. Production of the fibers from polymer solution provides the opportunity to introduce different additional compounds into electrospinning solutions like small molecules, biopolymers, sets of molecules, supramolecular structures or nanoparticles forming stable suspensions [24,25,26,27]. Moreover, electrospun scaffolds have already been used for DNA delivery alone or in supramolecular complexes [28,29,30,31,32]. It should be noted that the packaging of DNA in the fiber structure increases its stability in the organism for a very long time, reduces the immunogenic potential due to the low concentrations of the molecules, and ensures delivery to cells as a result of their contact with the materials. Moreover, packaging supramolecular complexes of DNA with transfection reagents in the fibers must decrease the toxicity of the latter, and thus reduce the local as well as general toxicity of DNA complexes to cells and tissues in vivo [29,33]. Previously, it was shown that the method of introducing DNA into fibrous materials and the methodology for their preparation influenced both DNA release and the level of cell transfection [34,35,36,37]. In most published studies, the authors demonstrated good transfection efficacy of such scaffolds, but noted a rapid release of the main plasmid DNA (pDNA) part, as well as a rather short period of total pDNA release [36,38]. Thus, such scaffolds are not suitable for reprogramming cells that inhabit the tissue engineering constructs, nor for maintaining their long-term ability to produce the proteins required. DNA strongly binds to the silica surface [39,40,41]. Therefore, the adsorption of DNA on the surface of silica nanoparticles can potentially decrease the DNA release rate due to DNA interaction with silica, and provide a constant particle size compared to supramolecular DNA complexes.

In this regard, we studied the gradual release of pDNA from electrospun scaffolds, produced such scaffolds loaded with nanoparticle (NP)-associated pDNA, and studied pDNA release as well as the transfection efficacy of pDNA into cells cultured on such materials.

## 2. Materials and Methods

### 2.1. pDNA Production

Plasmid DNA *phMGFP* (Promega, Fitchburg, WI, USA) was produced in Escherichia coli Stbl3 cells and isolated using an EndoFree Plasmid Giga kit (Qiagen, Hilden, Germany), which allows the pyrogen-free pDNA to be obtained. According to agarose gel electrophoresis, the pDNA contained no less than 90% of the supercoiled form, and contained no more than 20 EU lipopolysaccharide units per mg of pDNA, as found by the LAL test (CharlesRiver, Wilmington, NC, USA).

### 2.2. Preparation of Nanoparticles-Containing pDNA

The silica nanoparticles (NPs) were prepared as described in [42]. Briefly, 0.018 M tetraethoxysilane in ethanol was mixed with 25% ammonia to its final concentration of 0.75 M, and incubated at 25 °C for 12 h. The NPs were pelleted via centrifugation at 17,000× *g* for 7 min and washed with 96% ethanol, and then with water by centrifugation.

**Figure 1:**
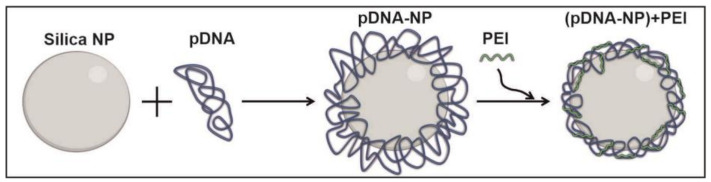
Schematic representation of the preparation of pDNA-coated silica nanoparticles.

The scheme for preparing the plasmid DNA-coated nanoparticles is shown in Figure 1. The NPs were activated according to the protocol described earlier to enhance DNA-binding capacity [39]. The binding of 4 µg of pDNA with 7 mg of particles was carried out in 500 µL of buffer containing 6.75 M GuSCN, 15 mM Tris-acetate, and 15 mM EDTA, at pH = 6.5 for 45 min at 25 °C. The pDNA-silica nanoparticle complexes (pDNA-NPs) were centrifuged for 10 min at 17,000× *g*, and the pellets were washed two times with 700 µL of buffer (10 mM Tris-HCl, 0.15 M NaCl, 75% ethanol, pH = 7.5) and two times with 75% ethanol via sedimentation of the nanoparticles. Then, the re-precipitated pDNA-NPs were suspended in 96% ethanol.

Complexes of pDNA-NPs with polyethyleneimine (PEI, M_W_ = 25 kDa) at N/P ratios = 3, 9 and 27 were obtained by mixing a corresponding volume of PEI with pDNA-NPs suspension, with subsequent incubation for 3 h at 25 °C, and washing out the non-bound PEI by centrifugation/precipitation of (pDNA-NPs) + PEI complexes three times in an excess of 96% ethanol.

The freshly prepared pDNA-NPs and (pDNA-NPs) + PEI complexes were used in all of the experiments.

### 2.3. Characterization of DNA-Nanoparticles

The silica NPs and pDNA-coated nanoparticles (pDNA-NPs and (pDNA-NPs) + PEI) were characterized via dynamic light scattering (DLS) on a Malvern Zetasizer Nano device (Malvern Instruments, Malvern, UK). The 3 samples of each nanoparticle type were used in DLS experiments.

Negative contrast staining for transmission electron microscopy was performed according to a previously described procedure [43], with modifications. In brief, fresh samples of particles (5 μL) were absorbed on formvar/carbon-coated and glow-discharged copper EM grids (Agar Scientific, Essex, UK) for 10 s. The grids were then stained for negative contrast with a drop of 0.5% uranyl acetate on parafilm for 2 min. The excess fluid was then removed using filter paper, and the grids were air dried. Finally, the samples were analyzed with a JEOL1400 microscope (JEOL, Tokyo, Japan) at 80 kV at the Center for Microscopy of Biological Subjects (Institute of Cytology and Genetics SB RAS, Novosibirsk, Russia). The size of the particles was evaluated using 10 TEM images (total number of particles no less than 30) for each type of the NPs.

### 2.4. Fabrication of Electrospun Scaffolds

The basic electrospinning solution contains a blend of 5% polycaprolactone (PCL) (*w*/*v*) with 10% gelatin (Gl) (*w*/*w* PCL) in hexafluorisopropanol (HFIP). Control scaffold was produced from the basic solution using an NF-103 (MESS, Fukuoka, Japan) electrospinning device under the following conditions: a collector rotation speed of 350 rpm, a voltage of 18.5–18.8 kV and a solution flow rate of 0.7–0.9 mL/h. The freshly prepared NPs in 100 µL of 96% ethanol (~12 mg) were dispersed in 1 mL of basic electrospinning solution for 1 h, in order to obtain a suspension (18% by weight of polymers). For X-ray photoelectron spectroscopy (XPS) and mechanical testing, scaffolds were produced only from suspensions containing basic solution with the addition of 18% (by weight) of nanoparticles in the aforementioned conditions. For the SEM and pDNA release studies, 2-layer scaffolds (to reduce pDNA consumption) were produced: an 80% thickness layer was electrospun from basic solution, whereas the remaining 20% thickness layer was electrospun from suspensions containing basic solution with 18% (by weight regarding the total weight of PCL plus Gl) of the different nanoparticle types.

### 2.5. Characterization of Electrospun Scaffolds

The morphology of the electrospun fibrous scaffolds was examined with a scanning electron microscope (SEM) (EVO 10, Carl Zeiss AG, Jena, Germany). The fiber diameter and pore size were assessed in SEM images by the SmartTiff program (Zeiss, München, Germany) according to ISO 7198:1998 [44].

Mechanical properties were measured using a Zwick/Roell Z10 (Northeim, Germany) universal testing machine with an elongation rate of 10 mm/min. At least 3 samples (50 mm × 10 mm, dog bone shape) per scaffold were tested to determine their ultimate tensile strength and ultimate elongation.

XPS was performed using a SPECS electron spectrometer equipped with a PHOIBOS-150 MCD-9 hemispherical analyzer and a non-monochromatic MgK source (SPECS GmbH, Berlin/Heidelberg Germany), as described previously [45].

### 2.6. In Vitro pDNA Release from Electrospun Scaffolds

The study was conducted in accordance with the Declaration of Helsinki, and approved by the Ethics Committee of ICBFM SB RAS (protocol code 1 and date of approval 15 January 2016).

To evaluate the pDNA release, discs from different scaffolds (diameter of 10 mm) were excised, placed in a 48-well plate and incubated with 500 µL of phosphate buffer (PBS) or EDTA-stabilized human blood plasma (HBP). The plate was sealed with a Microseal^®^ film (Bio-Rad, Hercules, CA, USA) to prevent drying and incubated on a Titramax 1000 shaker (Heidolph, Schwabach, Germany) at 37 °C with a 200 rpm platform rotation speed. Two series of pDNA release kinetics were studied. The materials were incubated in PBS or HBP for different time intervals, and then the supernatants were tested for the pDNA released (equilibrium conditions, C1). For dynamics conditions (C2), the supernatant was removed at each time point, then a fresh portion of PBS or HBP was added, and the discs were incubated in a fresh solution until the next time point, when the procedure of collecting/replacing the solution was repeated.

The pDNA from the obtained supernatants was isolated using the pDNA isolation Biosilica kit (Biosilica, Novosibirsk, Russia), according to the protocol recommended by the manufacturer. The concentration of pDNA was determined using real-time quantitative PCR at each time point of the kinetics study. The release profiles of pDNA from electrospun scaffolds were constructed considering the calibration curve obtained under the same isolation/RT-PCR conditions. The RT-qPCR experiments were performed on the real-time CFX96 touch system (Bio-Rad, USA) with hot-start HS-Taq polymerase (Biolabmix, Novosibirsk, Russia) and GFP-specific primers (Table 1).

### 2.7. Cultivation, Viability and Transfection Efficiency of Cells on Fibrous Scaffolds

A human embryonic kidney (HEK293T) cell culture was obtained from the Russian collection of cell cultures (Institute of Cytology RAS, Saint Petersburg, Russia). The monolayers were cultured in DMEM media containing 10% embryonic calf serum (Thermo Fisher Scientific, Waltham, MA, USA) and 100 u/mL of penicillin and streptomycin (Thermo Fisher Scientific, Waltham, MA, USA) in an atmosphere of 5% CO_2_ at 37 °C.

To study cell viability, adhesion, and transfection on scaffolds, discs from the scaffolds were prepared as described in Section 2.6, fixed with a Teflon o-ring at the bottom of a 48-well plate as described earlier [46], and then cells were seeded and cultivated as described above.

The viability of the HEK293T cells was tested using the Alamar Blue assay (Invitrogen, San Diego, CA, USA) after 2- and 5-day incubations of cells with scaffolds in 48-well plates. At the end of incubation, the growth medium was removed, the cells were washed with PBS, and fresh DMEM medium w/o phenol red supplied with 10% Alamar Blue was added into the wells for subsequent 4–6 h of incubation in a CO_2_ incubator [46]. Then, supernatants were transferred into 96-well plates, and optical extinctions at 570 nm versus 620 nm were measured using a Multiskan GO spectrophotometer (Thermo Scientific, Waltham, MA, USA). The experiments with cells were carried out in 3 technical replicates.

Cell adhesion on different scaffolds was studied using SEM. Samples with HEK293T were prepared for SEM analysis, as previously described [47]. Briefly, after 2 days of cultivation, the matrices were washed twice with PBS, fixed with 2% formaldehyde in a physiological saline solution for 30 min, and rinsed with PBS. The fixed cells were dehydrated using a graded ethanol series (50, 70, 96 and 100%) and incubated in ethanol/hexamethyldisilazane (HMDS) solution (in a ratio of 1:1), followed by incubation in 100% HMDS. To calculate the mean area of cells on the material, 30 random cells were analyzed using the Altami Studio 3.5 program (Altami Ltd., Saint Petersburg, Russia).

As a control of transfection efficacy, cells on a control scaffold (free of any pDNA) were transfected using a TurboFect transfection reagent and pDNA, according to the manufacturer’s protocol (Thermo Fisher Scientific, Waltham, MA, USA). A dose of 25 ng of pDNA per well was used in the transfection experiments.

The transfection level of the HEK293T cells cultivated with the pDNA-containing materials was studied with fluorescent microscopy and real-time quantitative PCR. For fluorescent microscopy and RT-qPCR, the cells were cultivated for 5 days on discs in 48-well plates. Then, for fluorescent microscopy, the cells were fixed in 2% formaldehyde in PBS and stained with Hoechst 33342 (Invitrogen, Waltham, MA, USA) and Phalloidin-TRITC (Sigma, Ronkonkoma, NY, USA), as previously described [48]. The samples were plated on microscopy slides (cells to the lens in fluorescent supporting medium), coated with cover glasses, and analyzed using a confocal laser scanning microscope LSM 780 NLO (Carl Zeiss, Jena, Germany) at 395 nm to detect GFP-protein, at 405 nm to detect cell nuclei, and at 543 nm to detect actin microfilaments, using ZEN 2 software (Carl Zeiss Inc., München, Germany).

To confirm the transfection efficacy via mRNA expression, the HEK293T cells cultivated on scaffolds were washed with PBS and lysed using 750 µL of TriZol reagent (Life Technology, Carlsbad, CA, USA) with subsequent RNA isolation, as described [49]. The RT reaction was executed in 20 µL containing 0.5 µg of RNA isolated from lysed cells, 100 pmol of d(T)_18_ primer and 50 U of M-MuLV reverse transcriptase, according to the manufacturer’s protocol (Biolabmix, Novosibirsk, Russia). The cDNA obtained was used in the qPCR experiments that were performed on a real-time CFX96 touch system (Bio-Rad, Hercules, CA, USA), with SYBR dye (Biolabmix, Novosibirsk, Russia), hot-start HS-Taq polymerase (Biolabmix, Novosibirsk, Russia), gene-specific primers, under the conditions listed in Table 1. The experiments were carried out in 3 technical replicates. The relative quantification of the GFP expression level was calculated versus constitutively expressed genes encoding GAPDH and 18S rRNA.

## 3. Results and Discussion

As we mentioned in the introduction, the delivery of DNA encoding specific genes from electrospun scaffolds may be a useful tool to drive the functioning of cells that penetrate artificial tissue-replacing scaffolds. Such patterns as the induction of cell migration, cell growth or inflammation-related factor secretion, as well as production of cell type conversion transcription factors, may be induced by pDNA transfection [11,50]. Considering the cell proliferation rate, which is characterized by the doubling time from 20 h to many days (depending on cell type), their lifetime and currently described DNA release kinetics from scaffolds occur incomparably faster. The task of prolonging the long-term DNA release for the support of long-time persistence in scaffolds of transfected cells is needed.

Actually, scaffolds produced by electrospinning from blends of pDNA with polymer solutions release pDNA rather quickly [34]. For example, pDNA polyplexes packed in biodegradable polymer fibers were shown to release 40–55% of their pDNA in 1 day, and residual amounts in 3–5 days of incubation [34]. It should be mentioned that the mechanisms of pDNA release were not studied, but this release cannot be explained by PLA hydrolysis, and will probably happen if less biodegradable materials are used for pDNA retention.

Nevertheless, some studies demonstrated longer release times of pDNA polyplexes from fibers obtained by emulsion electrospinning [36]. The formation of DNA polyplexes in the presence of PEI and PEG made it possible to achieve DNA release from electrospun scaffolds for 25 days, in contrast to scaffolds packed with DNA and PEI polyplexes alone. For the latter scaffolding, a rapid short-term release time was observed [36].

Previously, it was found that the method of introducing DNA into composite fibers based on poly(lactide-co-glycolide)/hydroxyapatite affects NA release from the scaffold in PBS [35]. A comparative study of three methods of introducing DNA (DNA coatings outside the fibers, DNA–chitosan particles outside the fibers and DNA–chitosan particles introduced into the solution for electrospinning) showed that in one case, 60% of the DNA was released after 3 days and 100% after 12 days of incubation in PBS. When DNA–chitosan particles were located outside the fibers, a slower release of DNA was observed; namely, 30 and 100% of the DNA were released after 3 and 27 days, respectively. The production of fibers from the solution/suspension containing DNA–chitosan particles that packaged the DNA in the fibers led to even slower DNA release: 16 and 100% of the DNA left the scaffolds after 3 and 60 days, respectively. A similar release pattern was observed for miRNA–silica nanoparticles from fibrous materials [51].

It should be mentioned that in many studies, the authors did not evaluate the kinetics of pDNA release, and did not study pDNA release in biological fluids like blood plasma from scaffolds, providing only data on the transfection efficiency [52,53].

In technical terms, the blending of pDNA polyplexes with some solvents used for electrospinning presents certain difficulties. For instance, the dissolution of pDNA–PEI complexes in HFIP leads to opalescence of the solution, as we found in the pilot experiments; this seems to be related the low solubility of DNA in HFIP. Indeed, flotation or the precipitation of suspensions and emulsions greatly complicates the ES process.

On the other hand, HFIP as a solvent for electrospinning solutions represents a lot of possibilities to mimic natural tissues, due to the solubility of proteins and the long list of synthetic polymers [45,48,54,55]. In this process, fibers produced from ES solutions of proteins with synthetic polymers in HFIP are usually covered with firmly bound proteins on their surface, thus mimicking the extracellular matrix and possessing increased biocompatibility as compared to synthetic polymers alone [48,49].

To summarize the aforementioned, one of the options for the realization of long-term pDNA release from scaffolds may be the sorption of DNA on nanoparticles and the packaging of such complexes into fibers. Thus, pDNA release rate is determined not only by DNA diffusion, but also by the dissociation of DNA from the nanoparticles. The low solubility of DNA in HFIP increases the stiffness of DNA in nanoparticle complexes, and prevents DNA loss (release) in ES solution. Since silicates readily bind to DNA and are widely used for DNA isolation [39,40,41], we used silica NPs as pDNA carriers for the production of long-term pDNA-releasing scaffolds. To produce these scaffolds, we used polycaprolactone as a basic polymer combined with 10% gelatin, in order to increase cell adhesion [56]. Previously, we demonstrated that drug-enriched PCL-based scaffolds are well suited for vascular stent coatings, demonstrating satisfactory mechanical properties and biphasic long-term drug delivery [57,58].

### 3.1. Synthesis and Characterization of silica NPs Containing pDNA

Silica-based nanoparticles have acceptable biocompatibility for biomedical applications [59,60]. They are susceptible to nucleophilic hydroxyl attack from the aqueous medium, which leads to hydrolytic destruction of the Si-O-Si bonds to orthosilicic acid, which is biocompatible and excreted with the kidneys [61]. For the particles used previously, the absence of toxicity was shown on cell lines A549 and HEK293FT in a sufficient concentration range up to 50 µg/mL [42], which is consistent with data obtained by other research groups [62,63].

The preparation of silica nanoparticles has previously been described in detail [42]. The NPs used in the present study have a hydrodynamic diameter of 52 ± 5 nm (ζ-potential of −2.26 ± 0.02 mV) with a value of polydispersity index = 0.09 ± 0.01, as shown with DLS (Table 2). The particle size measured via TEM coincides well with the hydrodynamic diameter, and is 56.3 ± 14.6 nm.

The conditions of DNA adsorption at the surface of silica nanoparticles and the interaction of pDNA and PEI were studied in pilot experiments. It was found that 1 mg of silica nanoparticles binds 1.5 µg of pDNA (data not presented). In all of the experiments, pDNA-NPs were obtained by the adsorption of 0.7 μg of pDNA per 1 mg of silica nanoparticles. (pDNA-NPs) + PEI complexes with different N/P ratios = 3, 9 and 27 were produced for the current study.

The characteristics of the pDNA-containing particles were determined via DLS and TEM (Table 2). The absorption of pDNA onto silica NPs leads to an almost five-fold increase in their hydrodynamic diameter as well as their ζ-potential (252 ± 45 nm and −10.7 ± 0.2 mV, respectively). At that time, the TEM data demonstrated only 1.5-fold growth in the particle size as compared to the initial NPs (77.9 ± 17.1 nm). At that point, the addition of PEI at N/P= 9 led to a decrease in particle size via TEM to 63.0 ± 9.7 nm, as well as in their hydrodynamic diameter and ζ-potential (205 ± 73 nm and −4.55 ± 0.3 mV, respectively) as compared to the pDNA-NPs.

The data obtained on the change in particle size indicates DNA adsorption at the surface of the NPs, whereas the introduction of PEI obviously led to DNA condensation, but did not lead to the dissociation of pDNA from the NPs. The increase in the ζ-potential during the formation of PEI complexes with DNA has been repeatedly described [64,65]. At the same time, the increase in the ζ-potential up to 30 EV is associated with the formation of large (up to 400 nm in diameter) complexes, in which the DNA is hidden deep inside. At equimolar concentrations of DNA and PEI, or at a slight excess of PEI, the complexes have a negative zeta potential, which is associated with the exposure of DNA on the surface of such complexes [64]. The formation of bulk complexes of DNA adsorbed on silica NPs with PEI is impossible, and, as follows from the ζ-potential, such complexes contain DNA close to the surface. In any case, the change in ζ-potential confirms pDNA adsorption as well as the formation of DNA–PEI complexes on the surface of the NPs.

It should be noted that parameters such as particle size are the main factors that determine the penetration of DNA complexes into the cell [66,67]. The small size of pDNA complexes is extremely important for detecting cell uptake in vitro [29,68], as well as in vivo diffusion in tissues [69]. Taking these considerations into account, the size of the particles obtained allows for efficient pDNA delivery into cells.

### 3.2. Properties of DNA-Enriched Electrospun Scaffolds

Naked and pDNA-modified NPs were used for the preparation of the ES suspension using a basic ES solution of PCL and gelatin in HFIP. The average effective density of silica nanoparticles for the range 40–200 nm measured with an aerosol particle mass analyzer varied between 1.7 and 1.9 g/cm^3^ [70], and was closer to 1.7 g/cm^3^ for the 50 nm NPs. Considering the HFIP density of 1.6 g/cm^3^ and basic ES viscosity of around 116 cP, the NP suspension in basic ES solution was rather stable, and no NP sedimentation was observed at least for 3 h, which exceeded the time required to produce scaffolding with electrospinning.

**Figure 2:**
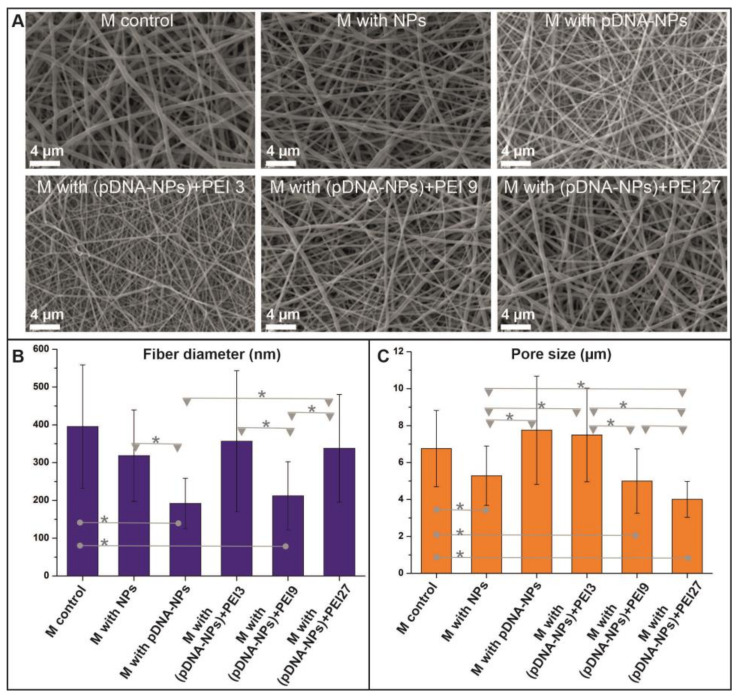
Structural parameters of studied scaffolds. (**A**)—SEM images of scaffolds. Scale bar is marked in white and represents 4 µm. (**B**)—Statistical analysis of fiber diameter for scaffolds (*p* < 0.05 is marked as *). (**C**)—Statistical analysis of pore size for scaffolds (*p* < 0.05 is marked as *).

The structure of the electrospun scaffolds was examined using SEM (Figure 2A). The control scaffold, which contained PCL with gelatin, had smoother surface fibers with a diameter of 395 ± 163 nm (Figure 2B). The composite scaffolds with NPs had rougher surface fibers that were associated with the distribution of particles at the fiber surface. For all of the scaffolds studied, the pore size varied in the range from 5 to 7.2 µm (Figure 2C). The introduction of different NPs in the polymer composition of the fibers led to a decrease in the fiber diameter in the 192 ÷ 356 nm (Figure 2B) range, which depended on the PEI content of the particles, and thus their charge.

The surface structure of the NPs-enriched scaffolds was studied using XPS (Table 3). Despite the introduction of 18% of silica NPs by weight (to the total weight of both polymers) in solution for electrospinning, the data of the Si2p spectra demonstrate that no more than 0.45% of Si was exposed at the fiber surface (carbon is considered 100%). Considering the surface charge of free NPs and the amino acid content of porcine skin Gl (2.7% Lys, 11% Arg, and 33.5% of nonpolar hydrophobic amino acids) [71], they were covered by a Gl and PCL layer whose thickness was no less than 10 nm (depth of element detection via XPS). These considerations looked reasonable, as TEM data demonstrated an approximately 10 and 6 nm layer of DNA or DNA-PEI complexes at the surface of the NPs. Given these data, it is not surprising that even less Si content was found on the surface of fibers with pDNA-NPs or (pDNA-NPs) + PEI. At the same time, we could not even find traces of phosphorous on the surface of the scaffolds. Indeed, the silica NPs in our experiments were loaded with no more than 0.07% of pDNA, and considering the detection of 0.45% of silicon at the surface of scaffolds, the phosphorous content must have been lower than the detection limit.

**Figure 3:**
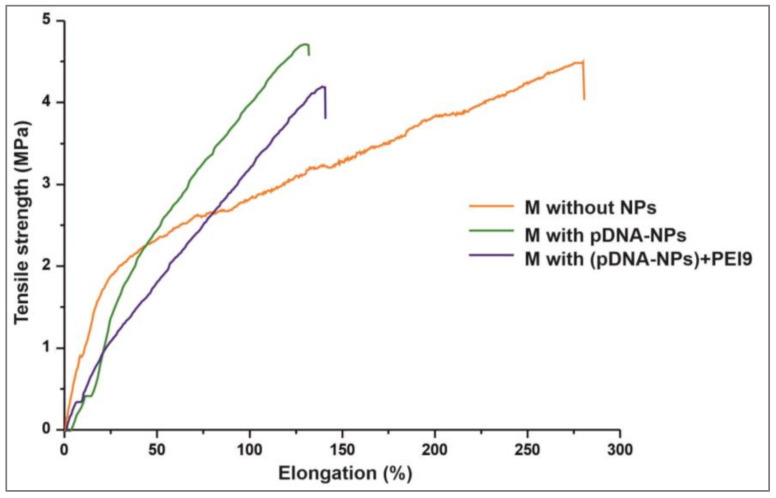
Typical tensile stress diagrams of studied scaffolds.

The typical tensile stress diagrams of electrospun scaffolds are presented in Figure 3. The introduction of NPs into the composition of polymer fibers led to a reduction in their ultimate elongation by approximately 2 times, from 280 ± 32% to 132 ± 27–141 ± 31%. However, the introduction of particles into the materials did not affect the values of the ultimate tensile strengths of the scaffolds, which varied from 4.18 ± 0.3 to 4.7 ± 0.48 MPa. Similar data were observed in the study of PCL materials without and with the addition of hydroxyapatite nanoparticles in poly(D,L-lactide) electrospun scaffolds [72,73]. At the same time, the silica NPs-enriched scaffolds were characterized by a lower Young’s modulus. These data are consistent with the data on the plasticization of polymer products and glues using physical fillers (particles), and their influence on the physical properties of particle-enriched polymer goods [74].

### 3.3. pDNA Release from Electrospun Scaffolds

**Figure 4:**
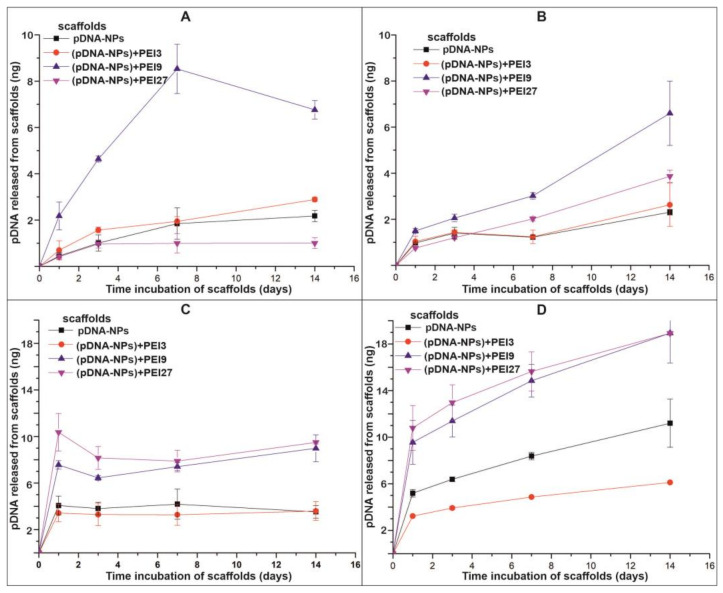
Kinetic curves of pDNA released from scaffolds. (**A**)—pDNA released in PBS in equilibrium condition (C1); (**B**)—pDNA released in PBS in dynamic conditions (C2); (**C**)—pDNA released in HBP in equilibrium conditions (C1); (**D**)—pDNA released in HBP in dynamic conditions (C2).

The release of pDNA from the scaffolds in PBS and HBP was studied over 14 days (Figure 4). When pDNA is released under dynamic conditions (C2), the data are presented in accumulating mode (previous time interval release plus current interval release). The percentage of pDNA released from the scaffolds was estimated using PCR, taking into account that the 10 mm discs from the electrospun scaffolds had a square area of 0.78 cm^2^ and contained ~175 ng of pDNA.

The pDNA released into the PBS can be described by two-phase kinetics: an initial fast release in the first day, with a subsequent slow release during the next 7 days in equilibrium conditions (C1) (Figure 4A), or after 7 days in dynamic conditions (C2) of the medium replacement (Figure 4B). It should be mentioned that in the period from 7 to 14 days of the (pDNA-NPs) + PEI9-incubated scaffolds with PBS in C1, the concentration of pDNA in the supernatant decreased, probably due to reabsorption of the DNA at the surface of the scaffolds. This phenomenon was not observed, or may not have been as pronounced for other scaffolds that contained pDNA. The maximum release under both conditions was characteristic of the scaffold with (pDNA-NPs) + PEI9, and made up ~4% of the pDNA introduced into the scaffold.

In order to determine the pDNA release mechanism from the studied scaffolds in PBS, the release data were fitted to the four kinetic models most commonly applied in release studies: the zero-order, first-order, Higuchi, and the Korsmeyer–Peppas models [75]. The highest values of the square of the correlation coefficient (r^2^) were detected for the Higuchi, the zero-Order and the Korsmeyer–Peppas models (Table 4).

The Korsmeyer–Peppas model describes drug release from polymeric drug delivery systems through a simple exponential relationship between fractional drug release and the release time: Mt/M∞ = kt^n^, where k is known as the transport constant. When drug release is governed by diffusion (Fickian release), then n will have a value of 0.5 (as in the Higuchi equation). On the contrary, n will be 1 for zero-order kinetics. Intermediate values of n (between 0.5 and 1) occur when mixed mechanisms of release are present. The release of pDNA from scaffolds containing pDNA-NPs or not strongly condensed (pDNA-NPs) + PEI3 complexes is obviously controlled by diffusion. At that point, the complexing of the DNA with NPs is not so important, at least when not too much time passes. The condensation of DNA by PEI on NPs leads to more complicated mechanisms of pDNA release from scaffolds, which better fit the Korsmeyer–Peppas model, which describes an exponential relationship between the fractional drug release and the release time. This variant of pDNA release is better suited to solving the problem of long-term maintenance of the required cell phenotype, and can be used to introduce drugs into scaffolds [76]. It should be mentioned that these models are effectively fitted for flat geometries, and may differ for cylindrical fibers packed in a scaffold [75].

The characteristic of pDNA release in human blood plasma is similar, but pDNA is released in HBP faster, especially during the first phase, with more pronounced reabsorption of pDNA on scaffolds when the scaffolds are incubated in C1 conditions (Figure 4C). The scaffolds with pDNA-NPs and (pDNA-NPs) + PEI3 released pDNA during the first day, and then up to day 14, the pDNA concentrations in their supernatants remained constant. The scaffolds with (pDNA-NPs) + PEI9 and (pDNA-NPs) + PEI27 during the first day released much more pDNA, as previously mentioned, and pDNA concentrations decreased to day 3, with subsequent growth to day 14. The maximum release under C1 conditions was characteristic of the scaffolds containing (pDNA-NPs) + PEI9 and (pDNA-NPs) + PEI27, with rates at about 5% of the pDNA introduced into the scaffold. Constant concentrations of the pDNA in HBP suggested an equilibrium between the pDNA released and pDNA remaining bound in the scaffolds, and indicate diffusion-dependent pDNA release from the scaffolds (Figure 4C).

The scaffolds demonstrated different pDNA release rates when incubated in HBP under dynamic conditions (C2). pDNA was released faster from scaffolds containing (pDNA-NPs) + PEI9 and (pDNA-NPs) + PEI27, whereas scaffolds with pDNA-NPs released pDNA less efficiently, and (pDNA-NPs) + PEI3 was even worse (Figure 4D). pDNA release under C2 conditions for scaffolds containing (pDNA-NPs) + PEI9 and (pDNA-NPs) + PEI27 reached ~11% of the total packed pDNA after 14 days of incubation. The difference in pDNA release under C2 conditions in HBP may be related to DNA binding to plasma proteins. Previously, it was shown that the bulk of plasma proteins bind to DNA fragments with affinities that vary in a range of 10^−5^–10^−8^ M^−1^ [77]. It should be mentioned that pDNA is released in PBS and HBP in a similar way to low-molecular-weight drugs packed in fibers [58,78]. Similarly to these drugs, pDNA is released into HBP in larger quantities and faster than in PBS. The binding of the drugs with HBP proteins and the decrease in the concentration of free drug in HBP were considered responsible for this drug release characteristic [76]. Probably, the pDNA-to-biopolymers interactions mimic pDNA complexes decreasing the concentration of initial pDNA–PEI complexes, and thus induce the release of pDNA in HBP.

After implantation, scaffolds inevitably come into contact with constantly renewed biological fluids. In dynamic conditions, scaffolds release approximately 10% of the introduced DNA in 14 days. Considering PCL scaffolds as rather stable [79,80] and taking into account the mechanism of DNA release, it can be predicted that DNA will be released in similar amounts for at least 3–4 months. This period is long enough for cell remodeling and reproduction to occur. Of course, this period may be shorter due to scaffold degradation under the pressure of cellular enzymes and active oxygen species [81], and the amount of pDNA released depends on the amount introduced into the fibers, including the long-term kinetics, which must be additionally studied.

### 3.4. Interaction of HEK293T Cells with Electrospun Scaffolds

In cells, pDNA delivery with the subsequent expression of encoded genes can be efficiently carried out in vitro, but is a more complicated task to achieve in vivo [82]. Actually, the biochemical market offers a number of reagents for pDNA transfection, like different Lipofectamine™ and GenJect™ [83,84], and a much shorter list for pDNA delivery in vivo [85,86]. Interaction with proteins and other biomolecules in body fluids, the toxicity of the transfection reagents in vivo and low concentrations of pDNA complexes complicate the delivery of pDNA in vivo [87].

Packaging the pDNA in a scaffold helps to overcome some of these problems. Actually, pDNA is delivered to cells located nearby, or directly to cells that are tightly bound with scaffolds. Thus, the concentration of pDNA complexes may be rather low, providing targeting pDNA delivery and preventing pDNA delivery into cells that are not closely bound with scaffolds, decreasing the problem of reagent toxicity. Being packed in scaffolds, the pDNA is hidden from nucleases presented in blood and other fluids in a reasonable concentration [88,89], and is prevented from binding to TLR9 receptors to result in undesirable biological effects [90,91,92]. Any extracellular matrix rearrangements (extracellular matrix degradation and remodeling) by cells due to the secretion of hydrolases and active oxygen species [93] may only be helpful for the delivery of pDNA packed in scaffolds, due to the increasing amount of pDNA complexes located near the cellular membrane. Thus, we studied the efficacy of pDNA transfection in cells cultivated on pDNA-enriched scaffolding.

Considering the change in the chemical composition of control scaffolds after the introduction of different types of NPs into fibers, we evaluated the interaction of HEK293T cells with scaffolds using cell viability tests and SEM.

**Figure 5:**
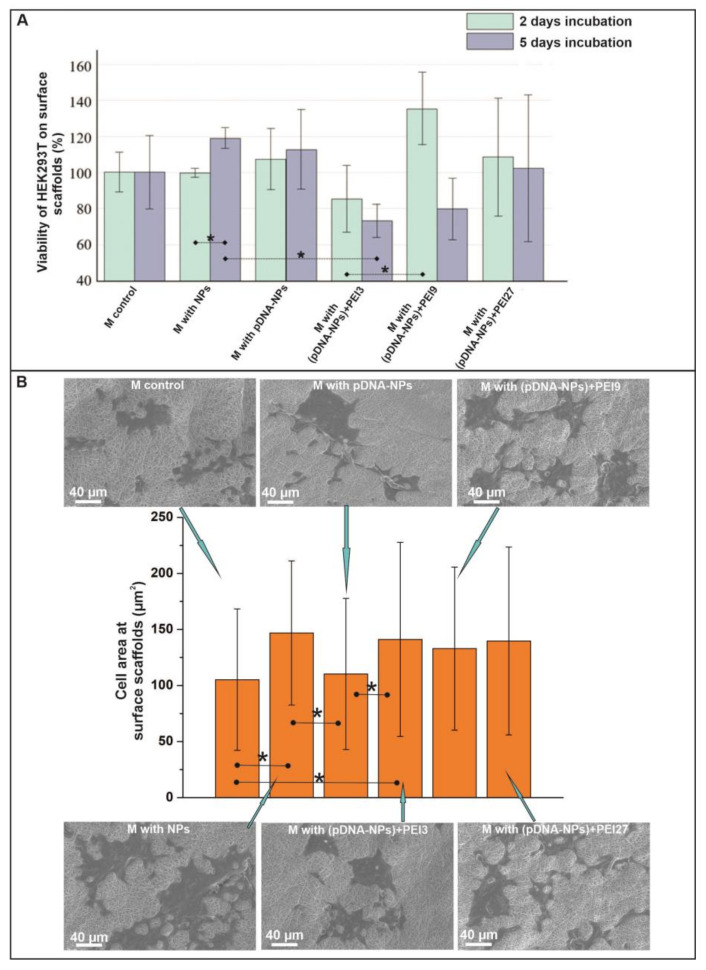
The interaction of HEK293T cells with the surfaces of studied scaffolds. (**A**)—Cell viability was tested with Alamar Blue assay after 2- and 5-day incubation of cells with scaffolds (Mean ± S.D. of three experiments). (**B**)—SEM images of cells on the surface of scaffolds after 2-day incubation. Cell area adhering to the surface materials (mean ± S.D.). * represents marked statistical significance of *p* < 0.05.

The data on the viability of HEK293T cells cultivated on surfaces of NPs-enriched scaffolds in comparison with the control scaffold after 2 and 5 days of incubation are presented in Figure 5A. It was found that the introduction of silica NPs in fibers had no effect on the viability of cells cultivated on such scaffolding. The data of the Alamar Blue test demonstrate that pDNA absorbed on NPs alone did not interfere with cell viability, whereas scaffolds containing pDNA-NPs treated with PEI demonstrated moderate cytotoxicity, which became more visible at a longer cultivation time (5 days). Similar data on the viability of human bone marrow stromal cells and human umbilical vein endothelial cells on materials containing pDNA–PEI complexes were recorded with a change in the amount of PEI in their composition after 1–7 days of incubation [52,94]. The SEM data representing a square occupied by cells demonstrated that cells readily spread on scaffolds with NPs containing the pDNA–PEI supramolecular complexes, the same as on scaffolds with silica NPs (Figure 5B). The control scaffold and scaffold with pDNA-NPs were less preferable for HEK293T cells in terms of cell adhesion and spreading. It should be noted that cell spreading is beneficial for pDNA delivery, as a larger cell contact area allows more DNA to enter cells.

**Figure 6:**
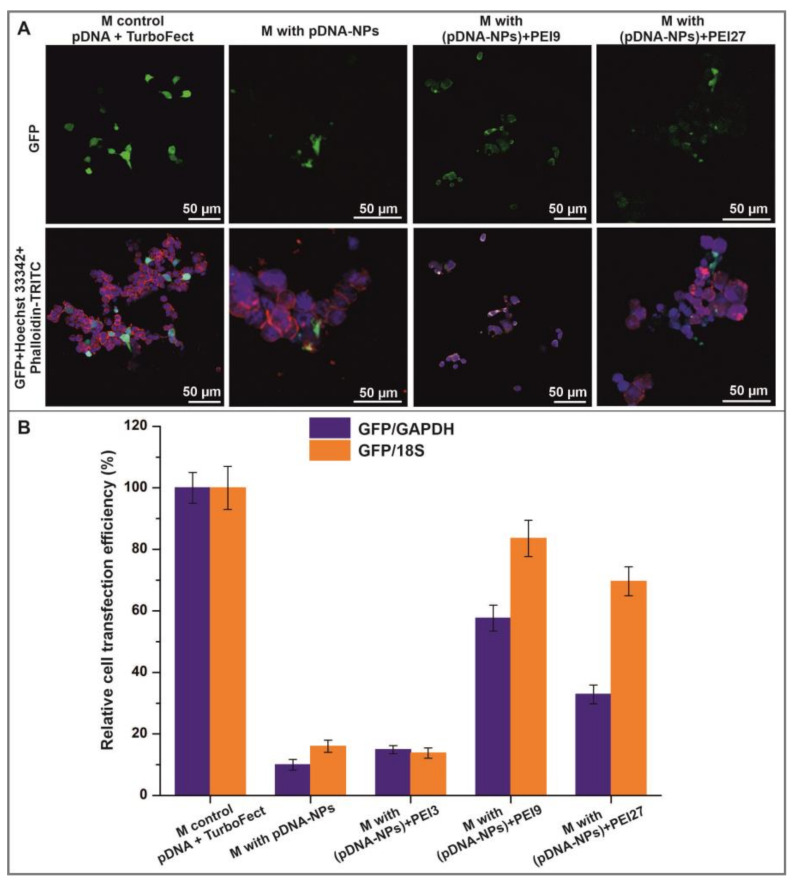
The transfection of HEK293T cells after 5 days of incubation with different scaffolds. (**A**)—Images of cells on surface scaffolds obtained by fluorescence microscopy. Scale bar is marked in white and represents 50 µm. (**B**)—Data obtained using qPCR (mean ± S.D. of three experiments).

According to the fluorescence microscopy data, a visible level of HEK293T cell transfection was noted when cells were cultivated on (pDNA-NPs) + PEI-enriched scaffolds compared with scaffolds that contained pDNA-NPs (Figure 6A). The fluorescent microscopy data were confirmed with RT SYBR Green qPCR. Better GFP expression was found on the scaffolds containing (pDNA-NPs) + PEI9 particles after 5 days of incubation (Figure 6B). The control cells were incubated on PCL-GL scaffolds and transfected with 25 ng of pDNA in a complex with TurboFect reagent. As compared to mRNA expression in pDNA TurboFect-transfected cells, the mRNA expression in cells cultivated on (pDNA-NPs) + PEI9 reached 57% (normalized to GAPDH), and even 82% when normalized to 18S (Figure 6B).

The transfection efficacy of cells cultivated on other pDNA-enriched scaffolds was less efficient, and scaffold containing (pDNA-NPs) + PEI9 is to be considered the most efficient pDNA transporter into cells. These data are consistent with the data from fluorescent microscopy on the delivery of pDNA from a DNA-enriched scaffold. The more intensive fluorescence observed in control cells transfected with TurboFect reagent could be related to an inhibition of cell growth on scaffolds enriched with pDNA and PEI, and better spreading of the cells on these scaffolds (increased cell square and fluorescence distribution on large square). It should be mentioned that the GFP versus 18S expression was significantly higher than GFP versus GAPDH, suggesting less efficient protein synthesis in these cells.

Similar results have been demonstrated using an electrospun scaffold for gene delivery from nanofibers for engineering various tissues [16,50,88]. It should be noted that it is rather difficult to compare the transfection results obtained using different materials, cells, and methods, since this process depends on cell–matrix integration.

Summarizing the obtained data, it can be concluded that scaffolds containing a combination of nanoparticles and PEI deliver pDNA to adhered cells. We showed that (pDNA-NPs) + PEI9 particles in the composition of electrospun scaffolds provide a sloping pDNA release in biological media, and are balanced between their toxicity and transfection efficiency against HEK293T cells. Such improved properties of materials may hopefully allow for the long-term regulation of cellular phenotypes in tissue repair with engineering instruments.

## 4. Conclusions

The engineering of a heterogeneous tissue usually requires a combination of biomaterials with spatially organized material composition and signaling molecules for cells. Given these requirements, electrospinning is considered a promising technology for the production of tissue engineering devices. In the present study we demonstrated a strategy for the incorporation of nanoparticle-associated pDNA—PEI supramolecular complexes into electrospun nanofibers for localized gene transfection. The electrospun-produced scaffolds were studied, as well as the release of pDNA and cell transfection. It was demonstrated that pDNA is slowly released from scaffolds and is able to transfect cells; thus, electrospun pDNA-enriched scaffolds can be used in tissue engineering and cell-based therapy. At this point, the long-term potentialities for pDNA transfection as well as its dependence on the reagents used and the amount of pDNA packed must additionally be studied.

## Figures and Tables

**Table 1:** Primer sequences and PCR conditions used in the study.

Gene	Primer Sequences (5′-3′)	PCR Conditions
*GFP*	F: CGTGTCTTCGCCAAGTAC R: CTTCATCATGGTGATGTCGT Probe:(Cy3)-CTCGTGGGAGC-(BHQ2)	95 °C for 3 min, 35 cycles 95 °C for 40 s, and 60 °C for 60 s.
*GAPDH*	F:TGAAGGTCGGAGTCAACGGATTTGGT R: CATCGCCCCACTTGATTTTGGAGGG	95 °C for 3 min, 40 cycles 95 °C for 20 s, 57 °C for 20 s, and 72 °C for 40 s.
*18S*	F: AAACGGCTACCACATCCAAG R: CAATTACAGGGCCTCGAAAG	95 °C for 3 min, 40 cycles 95 °C for 20 s, 60 °C for 20 s, and 72 °C for 40 s.

**Table 2:** Characterization of naked and DNA-modified NPs via DLS and TEM.

	Dynamic Light Scattering	TEM	*p* ^#^
Silica NPs	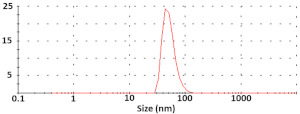 Diameter = 52 ± 5 nm ζ-potential = −2.26 ± 0.02 mV	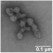 Size = 56.3 ± 14.6 nm	0.138
pDNA-NPs	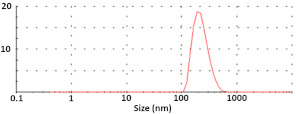 Diameter = 252 ± 45 nm ζ-potential = −10.7 ± 0.2 mV	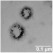 Size = 77.9 ± 17.1 nm	0.008
(pDNA-NPs) + PEI9	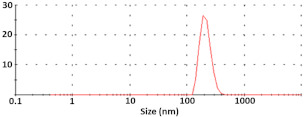 Diameter = 205 ± 73 nm ζ-potential = −4.55 ± 0.3 mV	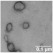 Size = 63.0 ± 9.7 nm	0.001
	*p*_1,2_ = 0.006; *p*_1,3_ = 0.006; *p*_2,3_ = 0.447	*p*_1,2_ < 0.001; *p*_1,3_ = 0.159; *p*_2,3_ = 0.099	

*p*^#^—Horizontal comparison for particle size obtained via DLS and TEM.

**Table 3:** Elemental analysis of the surface of various materials obtained using XPS *.

Scaffold	O	N	Si	P
M control	331.1	26.3	-	-
M with NPs	347.2	30.1	5.4	-
M with pDNA-NPs	351.0	35.1	1.4	-
M with (pDNA-NPs) + PEI9	344.4	38.3	3.4	-

* The content of elements was normalized to 1000 carbon atoms.

**Table 4:** The values of r^2^ for different kinetics models of pDNA released from scaffolds in PBS (C1).

Scaffold	Higuchi	Zero-Order	First-Order	Korsmeyer–Peppas
M with pDNA-NPs	0.9536	0.9536	0.8687	0.9715
M with (pDNA-NPs) + PEI3	0.9730	0.9730	0.9330	0.9655
M with (pDNA-NPs) + PEI9	0.6205	0.6205	0.4653	0.8253
M with (pDNA-NPs) + PEI27	0.5705	0.5705	0.4206	0.7261

## Data Availability

Data presented in this study are available on request, owing to privacy and ethical restrictions.
